# Comparison of physiochemical attributes, microbial community, and flavor profile of beef aged at different temperatures

**DOI:** 10.3389/fmicb.2022.1091486

**Published:** 2022-12-22

**Authors:** Haojie Yu, Songshan Zhang, Xiaochang Liu, Yuanhua Lei, Meng Wei, Yinchu Liu, Xiaodong Yang, Peng Xie, Baozhong Sun

**Affiliations:** ^1^Institute of Animal Sciences, Chinese Academy of Agricultural Sciences, Beijing, China; ^2^Chemical Engineering Institute, Shijiazhuang University, Shijiazhuang, China

**Keywords:** aged beef, temperature, tenderness, microbial community, vacuum packaged, flavor analysis

## Abstract

Beef aging for tenderness and flavor development may be accelerated by elevated temperature. However, little to no research has been undertaken that determines how this affects other important meat quality characteristics and microbial community. This study aims to decrease aging time by increasing temperature. Beef were aged and vacuum packaged at 10 and 15°C, and the effects of increased temperature on meat physiochemical attributes, microbial community, and flavor profile were monitored. The shear force decreased with aging in all temperature and showed the higher rate at elevated temperatures compare to 4°C. The beef aged at elevated temperatures (10 or 15°C) for 5 days showed equivalent shear force value to the beef aged at 4°C for 10 days (*p*  >  0.05), however, the final tenderness was not affected by the elevated temperature. The beef aged at elevated temperatures showed a significantly higher cooking loss and less color stability compared to 4°C (*p*  <  0.05). The total volatile basic nitrogen and aerobic plate count increased (*p*  <  0.05) faster at elevated temperatures compare to 4°C. Carnobacterium, Lactobacillus and Hafnia–Obesumbacterium were the dominant genus in the beef samples aged at 4, 10, and 15°C, respectively. In addition, the contents of isobutyraldehyde, 3-methylbutyraldehyde, 2-methylbutyraldehyde, and 3-methylbutanol were higher than aged at 4°C (*p*  <  0.05). Therefore, these results suggest that application of elevated aged temperatures could shorten required aging time prior while not adversely affecting meat quality. In turn, this will result in additional cost savings for meat processors.

## Introduction

Tenderness is the major eating quality of beef, and consumers are willing to pay a higher price for the beef that is guaranteed to be tender (Marino et al., 2013). In the beef industry, aging is widely used to improve tenderness, which can be affected by complex changes during muscle metabolism after slaughter (Colle and Doumit, 2017). The aging of beef is essential to provide a tender product and influenced by temperature. Beef were usually aged for 14 days at 4°C by processors (Kilgannon et al., 2019). However, this conventional aging temperature takes considerable refrigerated space requirements, operational losses, and energy (Wahlgren, 1994). Koohmaraie (1992) demonstrated that decreasing aging temperature reduced the autolysis of key muscle proteins and increasing aging temperature could improve tenderness. Consequently, aging temperature determined tenderization rates. When beef was aged at higher temperatures, beef tenderness improved largely within a shorter period (Kim et al., 2018). Pierson (1976) found that aging beef at an elevated temperature (20°C versus 3°C) for short periods (3–5 days) increasesd muscle tenderness. Meanwhile, Devine (1994) found that higher aging temperatures of approximately 10–15°C resulted in the highest degree of meat tenderness with the lowest muscle shortening and maximum aging potential.

Higher aging temperatures can increase beef tenderness within less aging time, meanwhile the proliferation of microorganisms in beef is promoted, which can lead to a significant reduction in shelf life (Zhu et al., 2004). Discoloration, off-odors, and slime formation caused by the deteriorative effects of microbial growth makes meat unacceptable to consumers (Esteves et al., 2021). Microbial growth is closely related to temperature. For example, total viable bacterial counts in beef stored at 10°C for 72 h increased by 2 log CFU/cm^2^, whereas those in beef stored at 5°C increased by 0.4 log CFU/cm^2^ (Kinsella et al., 2009). Moreover, Gribble et al. (2014a) reported that the counts of lactic acid bacteria (LAB), *Enterobacteriaceae*_,_ and *Brochothrix thermosphacta* increased with the prolongation of storage time regardless of the experimental temperature (−1.5, 0, 2, and 7°C). Vacuum packaging is often used to prolong the shelf life of beef, given that storage under anaerobic conditions proved to be very effective in extending the shelf life of various perishable foods (Mansur et al., 2019). Devine (1994) reported that temperatures of 10 and 15°C resulted in better tenderness of aged beef but the effect of higher aging temperatures on the color, microbial community, and flavor of beef were not evaluated.

This study aims to address the paucity of data on the effects of high temperatures on meat quality traits, microbial load, and flavor parameters and to provide a theoretical basis for shortening the aging time of beef.

## Materials and methods

### Sample preparation

*Longissimus dorsi* muscles were collected from six Simmental cattle (24 ± 1 months old, 470 ± 30 Kg) in a commercial abattoir and transferred to the laboratory in an ice box within 2 h. All beef were rinsed with sterile water to remove stains and blood, and then dried with sterilized paper towels. The muscles were cut into 300 g samples (6 cm × 6 cm × 10 cm) and vacuum packaged at the O_2_-transmission rate of 40 cm^3^/m^2^/day and 85% relative humidity. Samples were divided into three groups, and aged at respective temperature (4, 10, or 15°C). Samples were analyzed at different time points (0, 2, 4, 6, 8, 10, 14, and 18 day for 4°C, 1, 2, 3, 4, 5, 6, 8, 10, 12, and 14 day for 10°C; 0, 1, 2, 3, 4, 5, 6, and 7 days for 15°C). The temperature was monitored continuously by using remote temperature recorders with high precision control. Each analysis was performed using triplicate samples.

### Cooking loss and shear force measurement

The beef samples (3 cm × 3 cm × 6 cm) were packaged and cooked in a water bath at 80°C to achieve a core temperature of approximately 70°C. After cooking, the samples were cooled at 4°C until the core temperature cooled down to 10°C, surface-blotted with paper towels, and reweighed for weight loss. Cooking loss was determined by calculating the weight difference of the samples before and after cooking and expressed as the percentage of initial weight. Shear force values of samples (1.0 cm × 1.0 cm × 3.0 cm) weredetermined across the longitudinal direction of muscle fibers by a texture analyzer (TA-XT plus) attached with a Warner-Bratzler blade (V-notch, HDP/BSW). The cutting line was positioned to avoid fatty and connective tissues and was perpendicular to the muscle fiber direction. The shear force value was calculated as the mean of the maximum force and expressed as in Newtons (N).

### Instrumental color measurement

The surface color of the beef samples was measured on each analysis day after the samples were blooming for 30 min at room temperature. The lightness (L*), redness (a*), and yellowness (b*) of the beef samples were measured by a spectrophotometer (model CR-410, Minplta, Tokyo, Japan) with a diameter of 8 mm. The instrument was set for illuminant D65 and calibrated with a standard white plate before measurement. Measurements were taken in triplicate at different locations within each sample.

### Total volatile basic nitrogen measurements

Briefly, 10 g of raw meat (free of subcutaneous fat) was weighed into a closed glass vessel with 75 ml of distilled water with intermittent shaking for 30 min (room temperature). Immediately prior to distillation, 1 g of magnesium oxide was added to the sample, and the sample was steam distilled for 5 min. The distillate was condensed into a receiving flask containing boric acid (20 g/L) with a mixed indicator solution of bromocresol green/methyl red. The solution was back-titrated with 0.01 M hydrochloric acid solution, and TVB-N was calculated as mg/100 g raw meat.

### Microbiological analysis

A total of 25 g of samples from the top surface (depth: 0.5 cm) were blended in 225 ml of 0.85% sterile saline solution for 90 s in a stomacher (Ningbo Jiangnan Instrument Factory) at room temperature. Samples for microbial testing were prepared in a series of decimal dilutions by using sterile saline. Plate count agar (PCA) was used for total viable counts (TVCs). Violet red bile glucose agar (VRBA) was used for *Enterobacteriaceae* counts. Rogosa and Sharpe agar (MRS) was utilized for LAB counts. Cephalothin–sodium fusidate–cetrimide (CFC) agar with CFC-selective supplement was applied to determine *Pseudomonas* spp. counts. Streptomycin thallous acetate agar (STAA) with STAA selective supplement was employed for *Brochothrix thermosphacta* counts. Each diluent (0.1 ml) was spread on the selective medium agar in triplicate. CFC and STAA plates were incubated at 25°C for 48 h. PCA and MRS plates were incubated at 30°C for 48 h, and VRBA plates were incubated for 24 h at 37°C. The number of colonies was counted and expressed as colony forming units per gram (log CFU/g).

### Microbial-community analysis

#### DNA extraction and PCR

Total microbial genomic DNA was extracted from the beef samples using the E.Z.N.A.^®^ soil DNA Kit (Omega Bio-tek, Norcross, GA, United States) in accordance with the manufacturer’s instructions. The quality and concentration of DNA were determined using 1.0% agarose gel electrophoresis and a NanoDrop^®^ ND-2000 spectrophotometer (Thermo Scientific Inc., United States). The DNA samples were kept at −80°C prior to further use. The hypervariable V3–V4 region of the bacterial 16S rRNA gene was amplified with primer pairs 338F (5′-ACTCCTACGGGAGGCAGCAG-3′) and 806R (5′-GGACTACHVGGGTWTCTAAT-3′) using an ABI GeneAmp^®^ 9,700 PCR thermocycler (ABI, CA, United States). The PCR reaction mixture included 4 μl of 5 × Fast Pfu buffer, 2 μl of 2.5 mM dNTPs, 0.8 μl of each primer (5 μM), 0.4 μl of Fast Pfu polymerase, 10 ng of template DNA, and ddH_2_O to the final volume of 20 μl. The PCR amplification cycling conditions were as follows: initial denaturation at 95°C for 3 min, followed by 27 cycles of denaturation at 95°C for 30 s; annealing at 55°C for 30 s and extension at 72°C for 45 s; a single extension cycle at 72°C for 10 min; and a final cycle at 4°C. All samples were amplified in triplicate. The PCR product was extracted from 2% agarose gel and purified by using the AxyPrep DNA Gel Extraction Kit (Axygen Biosciences, Union City, CA, United States) in accordance with the manufacturer’s instructions and quantified using Quantus^™^ Fluorometer (Promega, United States).

#### Illumina MiSeq sequencing

Purified amplicons were pooled in equimolar amounts and paired-end sequenced on an Illumina MiSeq PE300 platform/NovaSeq PE250 platform (Illumina, San Diego, United States) in accordance with the standard protocols by of Majorbio Bio-Pharm Technology Co. Ltd. (Shanghai, China).

### Flavor analysis

The volatile compounds present in beef samples were identified and quantified by a GC–IMS flavor analyzer (FlavourSpec^®^, Shandong, China) equipped with a syringe and an autosampler unit for headspace analysis. Briefly, 2 g of homogenized beef sample was transferred into a 20 ml headspace vial, incubated at 60°C for 20 min, the vial was put in an incubator and shook at 500 rpm to facilitate the emitting of volatiles into headspace, the vial was sealed by using a magnetic cap with a silicone septum. The temperature of head space injection needle was 60°C and the injection volume was 500 μl. The analytical conditions for this test are as follows: Chromatographic column type: MXT-5, 15 m, 0.53 mmID, 1.0 μm df (RESTEK, United States), to separate the volatile components and coupled to ion mobility spectrometry (IMS); carrier/drift gas: N_2_, with the flow ramp starting at 2 ml/min for 2 min, then increasing to 20 ml/min in 8 min and increasing to 130 ml/min in10 min, finally 130 ml/min for 5 min, introducing the sample into the capillary; column syringe temperature: 85°C. The total GC runtime was 25 min, triplicate injections and analysis of samples were performed.

### Statistical analysis

All experiments were performed in triplicate. The mean values and standard errors of the means were recorded, outlying observations were identified, and implausible values were verified with the original source or coded as missing. Differences between treatments were analyzed through Tukey’s test. Statistical analyses were conducted at a 95% confidence level. Bioinformatics analysis was carried out on the microbiota by using the Majorbio Cloud platform on the basis of OTU information; rarefaction curves; and alpha diversity indices, including observed OTUs. The analytical software Laboratory Analytical Viewer and the built-in NIST and IMS databases of the GC–IMS Library Search software were used for the qualitative analysis of characteristic volatile compounds. One-way ANOVA was used to estimate the difference between means (*p* < 0.05).

## Results and discussion

### Cooking loss and shear force

**Figure 1:**
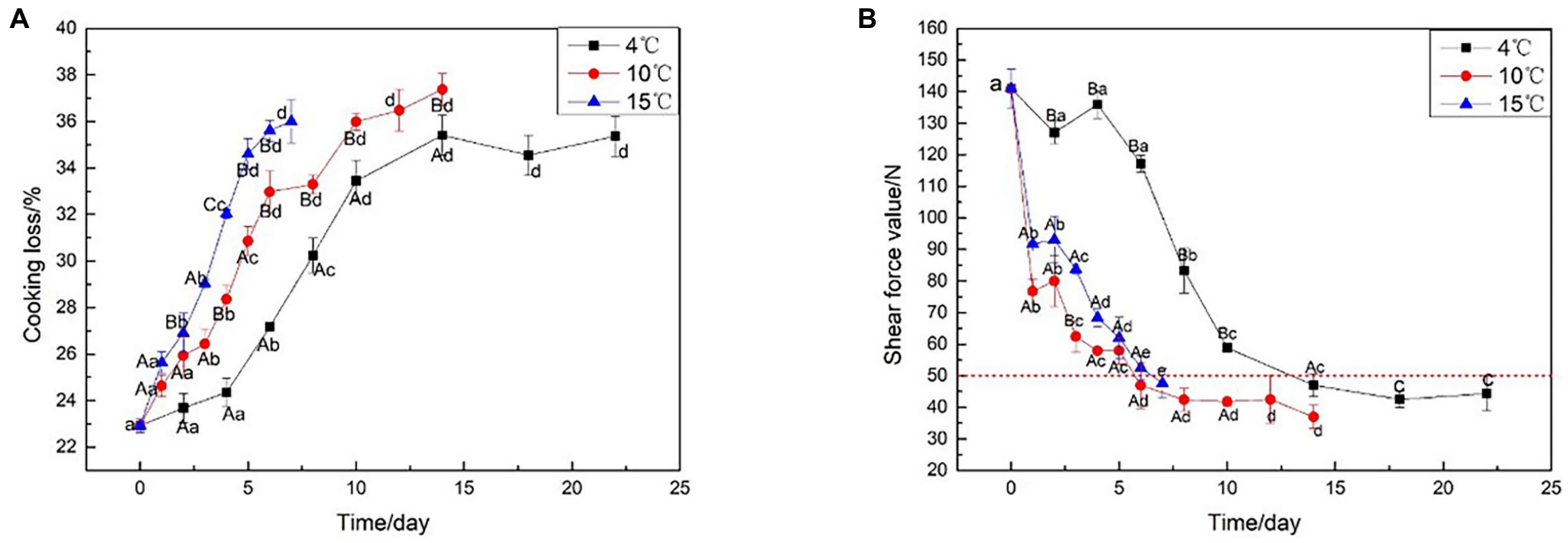
Cooking loss and Shear force analysis on beef samples aging at 4, 10, and 15°C. **(A)** Cooking loss, **(B)** Shear force analysis.

Cooking loss is the water loss from the meat due to protein denaturation during cooking (Kim et al., 2019). Figure 1A illustrates the effect of different aging temperatures on cooking loss, which tended to increase as the beef aged: the higher the aging temperature, the higher the cooking loss. In particular, the cooking loss aged at 15°C always higher than aged at 4°C (*p* < 0.05). Similarly, the interaction of aging temperature had an effect on the Warner-Bratzler shear force (WBSF) of the beef (*p* < 0.05). Figure 1B shows that with the prolongation of aging time, the shear force values show a decreasing trend whether it is decreased at 4, 10, or 15°C; specifically, aging affected the shear force values of all the beef samples with the meat becoming increasingly tender at each time point (*p* < 0.05). The beef aged at elevated temperatures (10 or 15°C) for 5 days had equivalent shear force values as the beef aged at 4°C for 10 days (*p* > 0.05). The beef aged at 15°C for 3 days had equivalent shear force values to beef aged at 4°C for 8 days (*p* > 0.05). This indicated that the aging period could be shortened by placing the meat at a slightly higher temperature than the typical meat aging temperature (4°C). However, the final tenderness is not affected by the elevated temperature. Aging at 4°C resulted in no significant reduction in shear force values before 6 days due to lower temperatures, however, the shear force values of the beef aged at 10 and 15°C decreased rapidly to 47.9 and 47.1 N, respectively, at the same stage of aging. At 14 days after aging at 4°C, the beef samples had an average WBSF value of less than 50 N. Aging at 10°C and 15°C required about 6 days, to achieve the same results, likely due to the enhancement in proteolytic enzyme activity, proteolysis *via* calpains, and collagen fiber breakdown from lysosomal enzyme activity at high temperatures (Kim et al., 2016). Furthermore, when the beef shear force under 50 N, the change is no longer significant. The time point could be considered as the end of beef aging, to save aging time and energy consumption.

### Surface color

**Figure 2:**
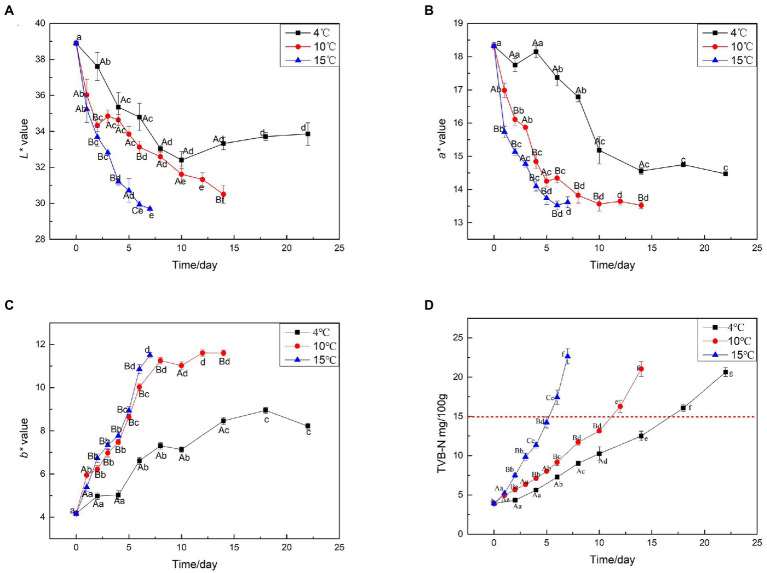
Surface color and TVB-N analysis on beef samples aging at 4, 10, and 15°C. **(A)** L* value, **(B)** a* value, **(C)** b* value, **(D)** TVB-N value.

Meat color is a direct estimate of meat freshness and wholesomeness for consumers, who often associate discoloration with spoilage (Li et al., 2015). The L*, a*, and b* color attributes of beef samples are shown in Figures 2A–C. The L* value decreased with the extension of aging time. Groups aged at higher temperatures presented significantly decreased L* values compared with those aged at 4°C. a* is the most important color parameter for fresh meat (Yim et al., 2016). Generally, the higher a* value, the fresher the meat. Figure 2B shows that a* decreased with the extension of aging time. At the same aging time, the a* values of samples aged at 10°C and 15°C were lower than those of samples aged at 4°C, indicating that the higher temperature, the more unfavorable a* value of beef. With the progression of aging, the b* value increased, and the b* value of samples aged at 15 and 10°C was significantly higher than that of the samples aged at 4°C (*p* < 0.05). Furthermore, aged at 4°C for 14 days had equivalent b* values as the beef aged at elevated temperatures (10 and 15°C) for 5 days, indicating that the higher temperature, the more unfavorable the effect on meat color.

### TVB-N analysis

TVB-N refers to the combined action of endogenous enzymes and bacteria in the muscle during the storage of animal food (Holman et al., 2021). It is highly temperature-sensitive and increases rapidly with small increases in storage temperature (Frank et al., 2019). The trend of TVB-N during aging at 4, 10, and 15°C was shown in Figure 2D. During aging at 4°C, the TVB-N content increased from the initial 4.6 to 12.5 mg/100 g after 14 days (*p* < 0.05). In vacuum-packaged beef aged at 4°C, it increased to 15 mg/100 g in approximately 16 days. During aging at 10°C, the TVB-N content increased to 13.4 mg/100 g after 10 days and continued to increase to 15 mg/100 g on the next day. The TVB-N content exceeded 15 mg/100 g after 5 days of aging at 15°C, indicating that the TVB-N values of beef increased rapidly at high temperatures, rose slowly in the early stages of storage, then rapidly increased.

### Microbial counts

The changes in the microbial counts of beef samples aged at different temperatures are shown in Table 1. Temperature appears to be the most important factor that influences the spoilage and safety of meat (Kennedy et al., 2005). Significant aging time and temperature interactions affected the TVC and growth of *Pseudomonas*, *Brochothrix thermosphacta*, LAB, and *Enterobacteriaceae* (*p* < 0.05). Initially, microorganisms were present at low levels. The TVC in unaged beef samples was 3.1 ± 0.01 log CFU/g, indicating that the sample is of good quality (Yang et al., 2018). In beef samples aged at 4°C, TVCs increased slowly at the beginning of aging but increased rapidly on day 6 and exceeded 5.8 log CFU/g by day 14, at which the shear force value reduced to less than 50 N. During aging at 10°C, the microbial counts exceeded 7.0 log CFU/g on day 10, and when the shear value started to drop below 50 N on day 6, the corresponding microbial count was 6.0 log CFU/g. Similarly, during aging at 15°C, the shear value of beef started below 50 N on day 5, corresponding to the microbial count of 6.3 log CFU/g that did not exceed 7.0 log CFU/g (Yang et al., 2016). A TVC total of 7.0 log CFU/g is recommended as the load limit at the end of the shelf life for red meat, According to the microbial colony counts, the shelf life of beef samples as the aged temperature increased. In order to the quality and safety of beef, it could be consider transferring the beef to 4°C storage when the shear value of beef is reduced 50 N to extend the shelf life, which is also our next research program. *Pseudomonas*, *Brochothrix thermosphacta*, *Enterobacteriaceae*, and *Lactobacillus* increased with a trend similar to the trend shown by the total number of colonies. LAB was the dominant population in the vacuum-packaged beef samples in this study. Similarly, Gribble et al. (2014b) LAB, *Enterobacteriaceae*, and *Pseudomonas* spp. populations increased when the beef was subjected to higher aging temperatures.

**Table 1:** Microbial counts of beef samples under different aging temperature.

Colony counts (lg CFU/g)	Temperature	Aging time(days)										
		0	1	2	3	4	5	6	8	10	14	18
Total viable counts	4°C	3.1 ± 0.01	/	3.4 ± 0.05	/	3.9 ± 0.09	/	4.4 ± 0.05	4.8 ± 0.02	5.4 ± 0.05	5.8 ± 0.07	6.9 ± 0.05
	10°C		3.9 ± 0.12	4.3 ± 0.08	4.7 ± 0.04	5.2 ± 0.03	5.8 ± 0.11	6.0 ± 0.05	6.7 ± 0.21			
	15°C		4.1 ± 0.11	4.9 ± 0.04	5.3 ± 0.32	5.9 ± 0.14	6.3 ± 0.26					
*Pseudomonas* spp	4°C	2.0 ± 0.08	/	2.7 ± 0.05	/	2.9 ± 0.06	/	3.2 ± 0.03	3.7 ± 0.06	4.2 ± 0.04	4.4 ± 0.03	4.6 ± 0.04
	10°C		2.7 ± 0.03	3.5 ± 0.05	4.2 ± 0.11	4.7 ± 0.09	5.3 ± 0.10	5.7 ± 0.11	5.9 ± 0.14			
	15°C		2.4 ± 0.11	3.4 ± 0.14	3.8 ± 0.05	4.5 ± 0.03	5.9 ± 0.15					
*B*. *thermosphacta*	4°C	2.7 ± 0.01	/	3.0 ± 0.05	/	3.5 ± 0.02	/	4.3 ± 0.05	4.7 ± 0.06	5.1 ± 0.03	5.8 ± 0.06	5.9 ± 0.04
	10°C		3.8 ± 0.14	4.4 ± 0.13	5.0 ± 0.05	5.2 ± 0.06	5.4 ± 0.08	5.8 ± 0.03	5.8 ± 0.10			
	15°C		3.1 ± 0.19	3.9 ± 0.06	4.7 ± 0.05	5.1 ± 0.23	5.7 ± 0.31					
Lactic acid bacteria	4°C	2.5 ± 0.03	/	2.8 ± 0.06	/	3.6 ± 0.11	/	4.6 ± 0.05	5.1 ± 0.04	5.5 ± 0.06	6.3 ± 0.05	6.6 ± 0.06
	10°C		3.5 ± 0.03	4.2 ± 0.07	4.9 ± 0.01	5.4 ± 0.02	5.5 ± 0.05	6.2 ± 0.03	6.5 ± 0.09			
	15°C		3.8 ± 0.14	4.3 ± 0.11	5.6 ± 0.16	6.3 ± 0.09	6.7 ± 0.03					
*Enterobacteriaceae*	4°C	1.4 ± 0.07	/	1.7 ± 0.06	/	1.9 ± 0.02	/	2.3 ± 0.05	2.4 ± 0.04	3.2 ± 0.08	3.7 ± 0.05	4.2 ± 0.05
	10°C		1.7 ± 0.10	2.2 ± 0.03	2.5 ± 0.06	3.2 ± 0.04	3.3 ± 0.27	3.5 ± 0.18	3.7 ± 0.07			
	15°C		2.3 ± 0.04	2.7 ± 0.22	3.3 ± 0.14	3.7 ± 0.17	4.1 ± 0.15					

### Bacterial flora analysis

#### Overall structural changes in beef bacterial communities

In this study, high-throughput sequencing technology was used to investigate the microbiota found in beef samples collected at 14 days of aging at 4°C (Day14_4), 6 days of aging at 10°C (Day6_10), and 5 days of aging at 15°C (Day5_15).

**Figure 3:**
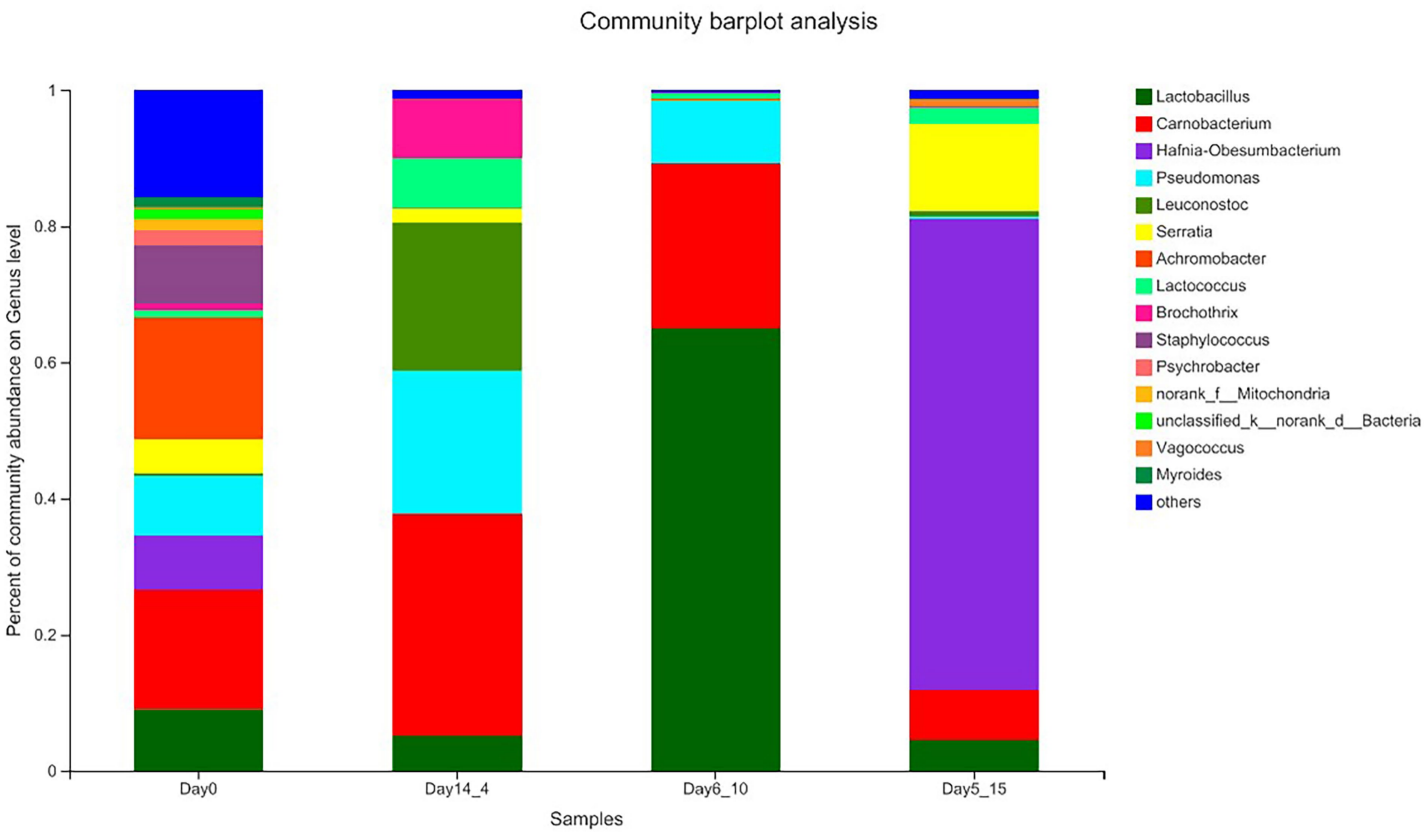
Relative abundance (%) of the top bacterial genera (Genus level) found on beef samples aging 14 days at 4°C, aging 6 days at 10°C, and aging 5 days at 15°C.

The results of the species annotations were as follows: domain: 1, kingdom: 1, phylum: 30, class: 69, order: 168, family: 269, genus: 454, species: 649, OTU: 974. The top five phylum included Firmicutes, Proteobacteria, Actinobacteriota, Bacteroidota, and unclassified_k__norank_d__Bacteria. The top five genera included *Lactobacillus*, *Carnobacterium*, *Hafnia–Obesumbacterium*, *Pseudomonas*, and *Achromobacter*. Figure 3 shows the percentage of the most abundant bacterial genera in Day14_4, Day6_10, 5 Day5_15 and 0 day beef samples because these points of time corresponded to shear values that started below 50 N. In the Day_0 sample, the dominant bacterial genera were *Vagococcus* and *Carnobacterium,* with average relative abundances of 17.95 and 17.60%, respectively, followed by *Lactobacillus*, *Pseudomonas*, *Staphylococcus*, *Hafnia–Obesumbacterium*, and *Serratia* with abundances ranging from 5.00 to 9.06%. *Leuconostoc*, *Brochothrix*, *Lactococcus*, and *Mitochondria* were also present in beef samples at abundances ranging from 0 to 5.00%. Chaillou et al. (2015) stated that these bacteria mainly originated from the meat processing environments, such as soil or water. In the sample aged at 4°C for 14 days, *Carnobacterium*, *Pseudomonas*, and *Leuconostoc* were the dominant genera. *Carnobacterium* increased to 32.55%, *Pseudomonas* increased from 8.78 to 21.01%, and *Cryptococcus* increased from 0.39 to 21.74% in the sample aged at 4°C for 14 days relative to those in the Day_0 sample. These species belong to the genus *Cryophilus* and exhibit good growth performance at low temperatures (Kaur et al., 2021). The genus *Lactobacillus* was clearly the dominant bacterial community in the Day6_10 sample and showed a seven-fold increase relative to that in the Day_0 sample and accounted for 65.02% of the total number of bacteria. This genus showed better growth performance at 10°C than at other temperatures. In the Day5_15 sample, the genus *Hafnia–Obesumbacterium* predominated, with its content reaching 69.24%, far exceeding the number of other genera. This result is a good indication that the structure of the flora in beef is affected by temperature. Low temperatures significantly favored the growth of LAB (*Carnobacterium* and *Leuconostoc*), whereas higher temperatures favored members of the phylum Proteobacteria (*Hafnia*). Given that the dominant genera often develop into specific spoilage bacteria, these results provide ideas for the precise prevention and control of spoilage bacteria in high-temperature aging.

#### Heatmap analysis of beef bacterial communities

**Figure 4:**
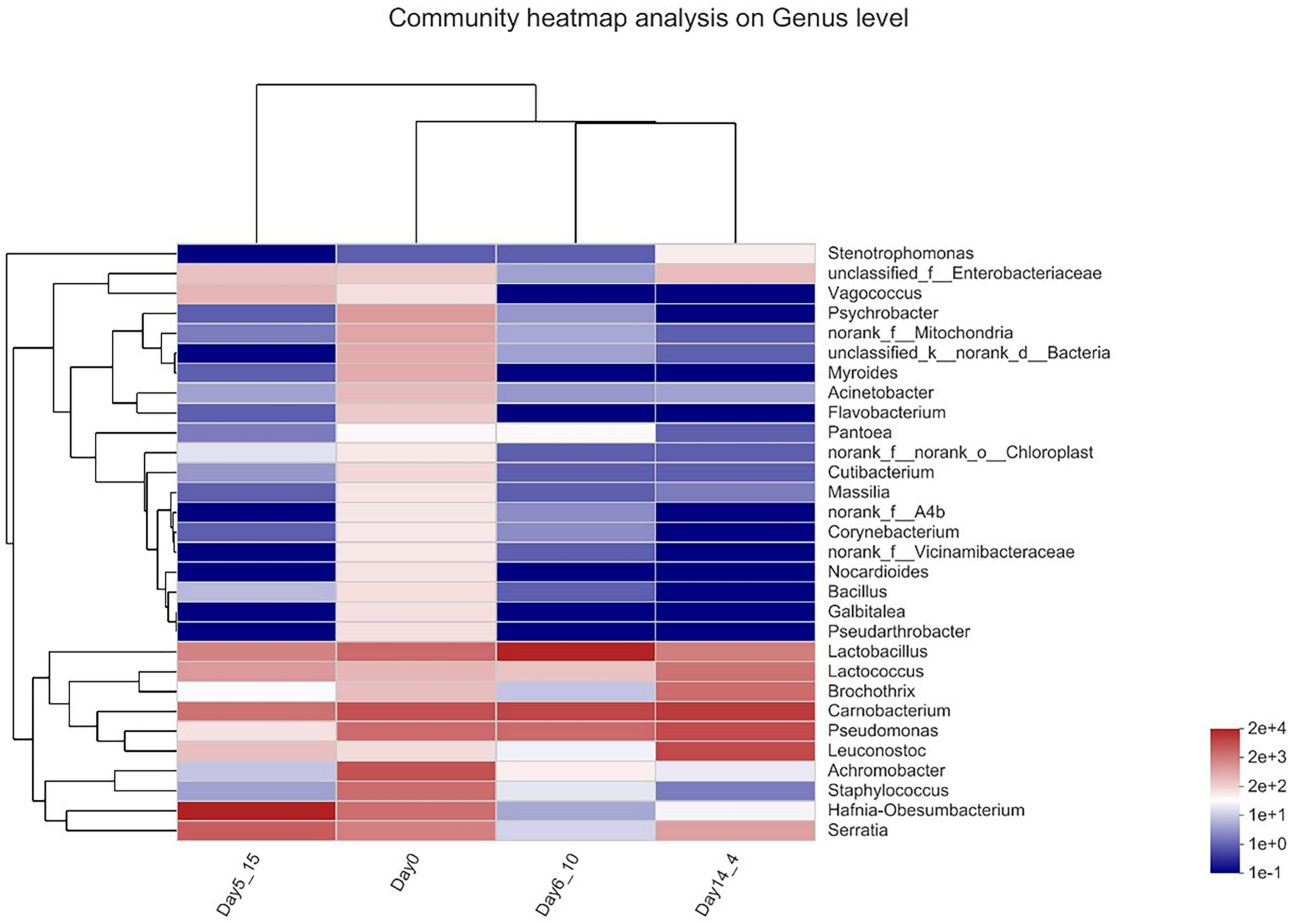
Heatmap of bacterial genera in beef samples found on beef samples aging 14 days at 4°C, aging 6 days at 10°C and aging 5 days at 15°C.

A genus-level clustering heatmap based on the top 30 genera in terms of relative abundance was constructed to analyze and compare the composition and dynamic changes in microbial communities in different samples (Figure 4). The horizontal coordinate is the sample name, the vertical coordinate is the genus name, and the color gradient of the color block shows the variation in the abundance of the different species in the sample, with the values represented by the color gradient on the right-hand side of the graph. The heatmap demonstrated that in the day 0 sample, *Carnobacterium* and *Vagococcus* clustered together with high relative OTU abundances during aging. *Carnobacterium*, *Pseudomonas*, and *Leuconostoc* clustered together with high relative OTU abundances in Day14_4. *Lactobacillus* clustered together with high relative OTU abundance on Day6_10 during aging. *Hafnia–Obesumbacterium* was present at higher relative contents in the Day5_15 sample. The bacterial compositions that gradually stabilized at late storage stages were generally dominated by bacteria that contribute largely to meat spoilage (Nychas et al., 2008).

### Characteristic volatile fingerprints

**Figure 5:**
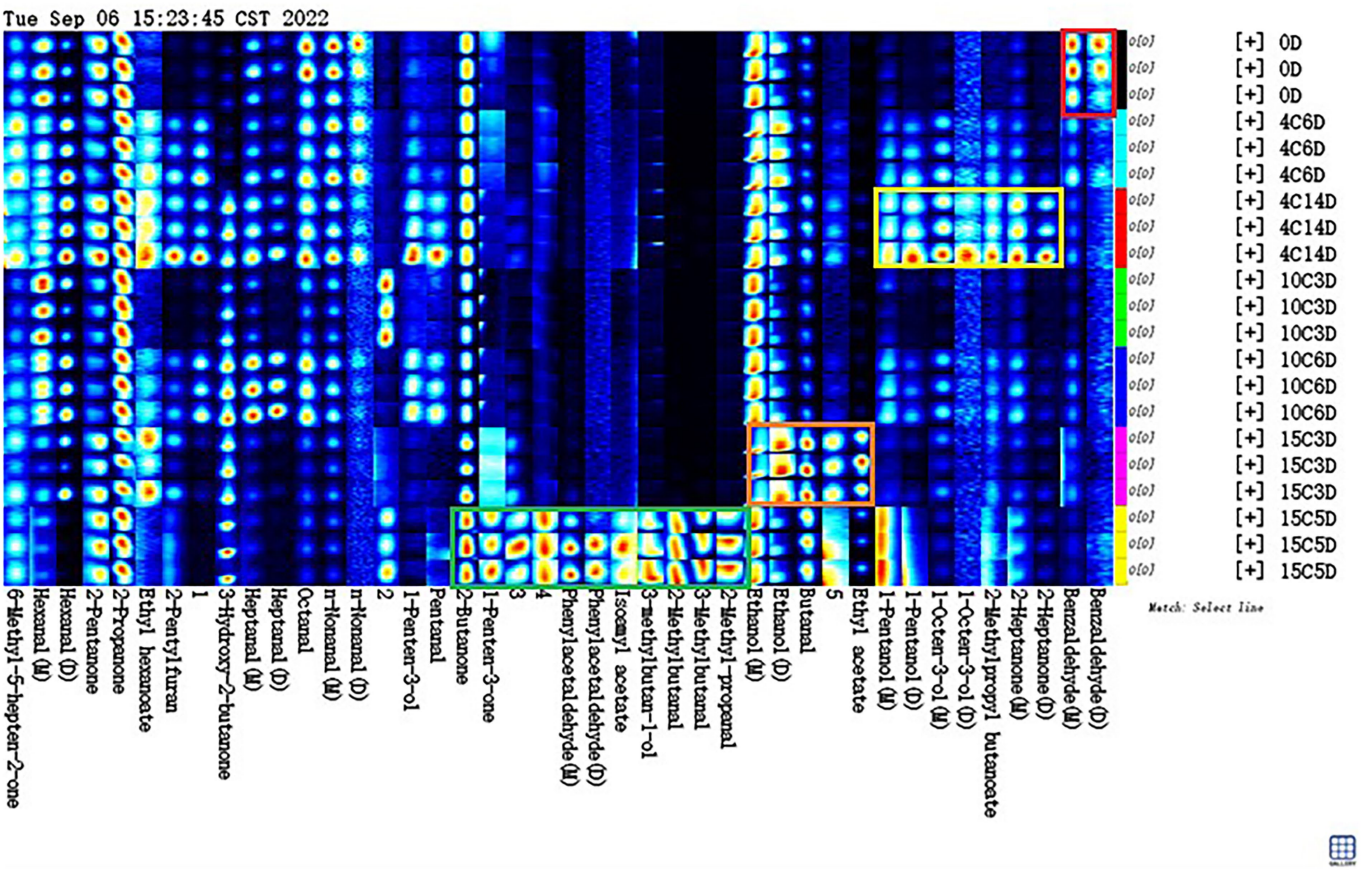
Fingerprint spectra of volatile compounds in beef samples different stages aging at 4, 10, and 15°C.

A characteristic fingerprint corresponding to each aging temperature was established by using gallery plots, and the difference and specific distribution of volatile compounds between different aging temperatures were compared intuitively (Figure 5). The Gallery Plot plugin of LAV software was used to compare the differences in the volatile compounds in beef samples at aged at different temperatures comprehensively. The colors of the plots indicated the signal strengths of the compounds: the darker the signal, the weaker the signal intensity and vice versa (Huang et al., 2022). The signal intensities of most volatile compounds significantly increased after aging at high temperatures, indicating that the levels of most volatile compounds were positively correlated with the aging temperature.

As shown in Figure 5, all the peaks to be analyzed in the obtained two-dimensional GC–IMS spectrum were automatically generated on the basis of fingerprints. Each row in the figure represents all signal peaks selected in a beef sample, and each column in the figure represents the signal peaks of the same volatile organic compounds in different beef samples. The substances in the red box in the picture had the highest content on 0 day compared to other groups. This substance included benzaldehyde, which decreased during aging at 4, 10, or 15°C. Benzaldehyde, is found in hyacinth, lemongrass, and rockrose and is a volatile compound that potentially results from the Strecker degradation of tyrosine, which has a bitter almond, cherry, and associated with a strong almond odor (Ma et al., 2012), if the level are raised too high, the undesirable flavor may result. The content of the substances in the yellow box in Figure 5 increased significantly in the 4°C_14D sample (*p* < 0.05). These substances included 2-heptanone, isobutyl butyrate, 1-octen-3-ol, and 1-pentanol. 2-Heptanone can produce a buttery, creamy, and cheesy aroma; it can be used as a spice raw material and is critical to aging flavor (Resconi et al., 2012). Isobutyl butyrate exists in pineapples and other fruits naturally and has a pleasant aroma. It is potentially a characteristic compound of fresh beef. 1-Octen-3-ol is positively correlated with umami flavor and is a volatile alcohol with a mushroom-like aroma that is found in dry-cured ham and dry sausage (Tansawat et al., 2013). Positive attributes, such as umami and juicy flavors in grain-fed steaks, are correlated with 1-octen-3-ol (Song et al., 2011). 1-Pentanol has a strong, sweet, balsamic aroma (Liu et al., 2022). Meanwhile, the dominant bacterial community was *Carnobacterium*, including that could produce these VOCs at low temperatures. The substances in the orange box in Figure 5 have the highest content in the 15C_3D sample. They included butanal and ethanol. Butanal is found in many essential oils, such as flowers, fruits, and dairy products, and has an ethereal fragrance when extremely diluted (Lee et al., 2019). Ethanol is the main component of wine, ethanol can also be used to make acetic acid, beverages, baked goods, and it generally have a low odour threshold, and thus they partly contribute to the flavour of cooked beef, what’s more, butanal and ethanol was found to decrease or remain stable at chill temperatures, whilst an increase was observed at elevated temperatures in vacuum packaged conditions. The substances in the green box in Figure 5 have the highest content in the 15C_5D sample compare to other groups. They included 2-methylbutanal, 3-methylbutanal, 3-methylbutanol, 2-methyl-propanal, isoamyl acetate, phenylacetaldehyde and 1-penten-3-one, and 2-butanone. 2-Methylbutanal is described as having a brothy, grainy or boiled meat aroma, which improves the sensory value of products (Lee et al., 2021). 3-Methylbutanal is a colorless and transparent liquid that is used as an intermediate in the production of flavors (Mansur et al., 2019). 2-Methylbutanal and 3-methylbutanal are Strecker degradation products of isoleucine and leucine, respectively (Saraiva et al., 2014). 2-Methyl-propanal is used in the synthesis of cellulose esters and flavors and is commonly applied in baked goods and meat products (Utama et al., 2018). Aldehydes, in general, are unstable and can easily react with other compounds to produce compounds with different flavors, it have a low odor-detection threshold; hence, even a trace amount can contribute to meat flavor, and, consequently, they are the most interesting of the lipid-derived volatiles (Legako et al., 2016). Isoamyl acetate has banana and pear aromas and is widely used in the production of various fruity edible flavors (Ercolini et al., 2009). Phenylacetaldehyde is naturally found in chicken, bread, rose oil, and citrus oil and confers beef with a clear and evocative aroma that differs in accordance with quality grade; specifically, it is higher in prime and low-choice steaks than in standard steaks (Zhu et al., 2016). 1-Penten-3-one is mainly used as a spice for food and in onion, garlic, and mustard flavoring; it has been identified as the main contributor to the integral flavor of beef due to its high odor activity values (Vilar et al., 2022). 2-Butanone is an intermediate in the preparation of pharmaceuticals, fragrances, antioxidants, and certain catalysts (Pavlidis et al., 2019). Meanwhile, the dominant bacterial community was *Hafnia–Obesumbacterium*, which can use the nutrients in beef to produce these compounds (Argyri et al., 2015). It could conclude that different aging temperatures produce different flavor profiles, on the one hand, these compounds could be formed protein degradation or oxidation of fatty acids; on the other hand, some bacteria studied in this work have probably contributed to different levels in the accumulation/depletion of the measured metabolic compounds.

## Conclusion

The results suggested that high-temperature (10 and 15°C) aging had a significantly shorter aging time than conventional aging (4°C) without affecting the safety of the product. This statement is supported by the practical equivalence of the surface color, TVB-N content, microbial counts, and flavor traits of the beef samples aged at high temperatures to those of the control samples. In this study, combining the total number of colonies and shear force values, it could be suggested that the aging time should be set 6 days at 10°C or 5 days at 15°C. On the basis of this new information, the beef industry is recommended to adopt shorter aging periods using slightly elevated temperatures 10 °C or and 15°C to capitalize on the advantages of a condensed aging period.

## Data availability statement

The raw data supporting the conclusions of this article will be made available by the authors, without undue reservation.

## Author contributions

BS, SZ, and XL designed the study. MW, YLe, and XY carried out the sample processing. HY performed the experimental data analysis and wrote the manuscript. All authors have read and agreed to the published version of the manuscript. All authors contributed to the article and approved the submitted version.

## Funding

This work was financially supported by the National Natural Science Foundation of China (No. 32072143), the Major Public Welfare Projects of Henan Province (No. 201300111200), the Agriculture Research System of China (No. CARS-37), the Special Basic Research Fund for Central Public Research Institutes (No. 2021-YWF-ZYSQ-07), and Science and Technology Plan Project of Tibet Autonomous Region (XZ202101YD0019C).

## Conflict of interest

The authors declare that the research was conducted in the absence of any commercial or financial relationships that could be construed as a potential conflict of interest.

## Publisher’s note

All claims expressed in this article are solely those of the authors and do not necessarily represent those of their affiliated organizations, or those of the publisher, the editors and the reviewers. Any product that may be evaluated in this article, or claim that may be made by its manufacturer, is not guaranteed or endorsed by the publisher.

## References

[ref1] Argyri A. A. Mallouchos A. Panagou E. Z. Nychas G. E. (2015). The dynamics of the HS/SPME–GC/MS as a tool to assess the spoilage of minced beef stored under different packaging and temperature conditions. Int. J. Food Microbiol. 193, 51–58. doi: 10.1016/j.ijfoodmicro.2014.09.020, PMID: 25462923

[ref2] Chaillou S. Chaulot-Talmon A. Caekebeke H. Cardinal M. Christieans S. Denis C.. (2015). Origin and ecological selection of core and food-specific bacterial communities associated with meat and seafood spoilage. ISME J. 9, 1105–1118. doi: 10.1038/ismej.2014.202, PMID: 25333463 PMC4409155

[ref3] Colle M. J. Doumit M. E. (2017). Effect of extended aging on calpain-1 and -2 activity in beef longissimus lumborum and semimembranosus muscles. Meat Sci. 131, 142–145. doi: 10.1016/j.meatsci.2017.05.014, PMID: 28527364

[ref4] Devine W. T. (1994). Effect of rigor temperature on muscle shortening and tenderisation of restrained and unrestrained beef m. longissimus thoracicus et lumborum. Meat Sci. 51, 61–72. 10.1016/s0309-1740(98)00098-9 22061537

[ref5] Ercolini D. Russo F. Nasi A. Ferranti P. Villani F. (2009). Mesophilic and psychrotrophic bacteria from meat and their spoilage potential in vitro and in beef. Appl. Environ. Microbiol. 75, 1990–2001. doi: 10.1128/AEM.02762-08, PMID: 19201980 PMC2663181

[ref6] Esteves E. Whyte P. Mills J. Brightwell G. Gupta T. B. Bolton D. (2021). An investigation into the anaerobic spoilage microbiota of beef carcass and rump steak cuts using high- throughput sequencing. FEMS Microbiol. Lett. 368, 1–10. doi: 10.1093/femsle/fnab109, PMID: 34472614

[ref7] Frank D. Zhang Y. Li Y. Luo X. Chen X. Kaur M.. (2019). Shelf life extension of vacuum packaged chilled beef in the Chinese supply chain. A feasibility study. Meat Sci. 153, 135–143. doi: 10.1016/j.meatsci.2019.03.006, PMID: 30933852

[ref8] Gribble A. Mills J. Brightwell G. (2014a). The spoilage characteristics of *Brochothrix thermosphacta* and two psychrotolerant Enterobacteriaceae in vacuum packed lamb and the comparison between high and low pH cuts. Meat Sci. 97, 83–92. doi: 10.1016/j.meatsci.2014.01.006, PMID: 24548927

[ref9] Gribble A. Mills J. Brightwell G. (2014b). The spoilage characteristics of *Brochothrix thermosphacta* and two psychrotolerant Enterobacteriacae in vacuum packed lamb and the comparison between high and low pH cuts. Meat Sci. 97, 83–92. doi: 10.1016/j.meatsci.2014.01.006, PMID: 24548927

[ref10] Holman B. W. B. Bekhit A. E. A. Waller M. Bailes K. L. Kerr M. J. Hopkins D. L. (2021). The association between total volatile basic nitrogen (TVB-N) concentration and other biomarkers of quality and spoilage for vacuum packaged beef. Meat Sci. 179:108551. doi: 10.1016/j.meatsci.2021.108551, PMID: 34000612

[ref11] Huang Q. Dong K. Wang Q. Huang X. Wang G. An F.. (2022). Changes in volatile flavor of yak meat during oxidation based on multi-omics. Food Chem. 371:131103. doi: 10.1016/j.foodchem.2021.131103, PMID: 34537608

[ref12] Kaur M. Williams M. Bissett A. Ross T. Bowman J. P. (2021). Effect of abattoir, livestock species and storage temperature on bacterial community dynamics and sensory properties of vacuum packaged red meat. Food Microbiol. 94:103648. doi: 10.1016/j.fm.2020.103648, PMID: 33279073

[ref13] Kennedy J. Jackson V. Blair I. S. McDowell D. A. Cowan C. Bolton D. J. (2005). Food safety knowledge of consumers and the microbiological and temperature status of their refrigerators. J. Food Prot. 68, 1421–1430. doi: 10.4315/0362-028x-68.7.1421, PMID: 16013380

[ref14] Kilgannon A. K. Holman B. W. B. Mawson A. J. Campbell M. Collins D. Hopkins D. L. (2019). The effect of different temperature-time combinations when ageing beef: sensory quality traits and microbial loads. Meat Sci. 150, 23–32. doi: 10.1016/j.meatsci.2018.11.023, PMID: 30562640

[ref15] Kim M. Choe J. Lee H. J. Yoon Y. Yoon S. Jo C. (2019). Effects of aging and aging method on physicochemical and sensory traits of different beef cuts. Food Sci. Anim. Resour. 39, 54–64. doi: 10.5851/kosfa.2019.e3, PMID: 30882074 PMC6411244

[ref16] Kim Y. H. B. Kemp R. Samuelsson L. M. (2016). Effects of dry-aging on meat quality attributes and metabolite profiles of beef loins. Meat Sci. 111, 168–176. doi: 10.1016/j.meatsci.2015.09.008, PMID: 26437054

[ref17] Kim S. Y. Yong H. I. Nam K. C. Jung S. Yim D. Jo C. (2018). Application of high temperature (14°C) aging of beef M. semimembranosus with low-dose electron beam and X-ray irradiation. Meat Sci. 136, 85–92. doi: 10.1016/j.meatsci.2017.10.016, PMID: 29107867

[ref18] Kinsella K. J. Prendergast D. M. McCann M. S. Blair I. S. McDowell D. A. Sheridan J. J. (2009). The survival of *Salmonella enterica* serovar Typhimurium DT104 and total viable counts on beef surfaces at different relative humidities and temperatures. J. Appl. Microbiol. 106, 171–180. doi: 10.1111/j.1365-2672.2008.03989.x, PMID: 19054240

[ref19] Koohmaraie M. (1992). Effect of pH, temperature, and inhibitors on autolvsis and catalvtic activitv of bovine a skeletal muscfe μ-calpain. J. Anim. Sci. 1:2. 10.2527/1992.70103071x 1429283

[ref20] Lee J. Featherstone A. Nayga R. Han D. (2019). The long-run and short-run effects of ethanol production on U.S. beef producers. Sustainability 11:1685. doi: 10.3390/su11061685

[ref21] Lee D. Lee H. J. Yoon J. W. Kim M. Jo C. (2021). Effect of different aging methods on the formation of aroma volatiles in beef strip loins. Foods 10:146. doi: 10.3390/foods10010146, PMID: 33445674 PMC7828147

[ref22] Legako J. F. Dinh T. T. N. Miller M. F. Adhikari K. Brooks J. C. (2016). Consumer palatability scores, sensory descriptive attributes, and volatile compounds of grilled beef steaks from three USDA quality grades. Meat Sci. 112, 77–85. doi: 10.1016/j.meatsci.2015.10.018, PMID: 26555563

[ref23] Li S. Zamaratskaia G. Roos S. Båth K. Meijer J. Borch E.. (2015). Inter-relationships between the metrics of instrumental meat color and microbial growth during aerobic storage of beef at 4°C. Acta Agric. Scand. Sect. A Anim. Sci. 65, 97–106. doi: 10.1080/09064702.2015.1072579

[ref24] Liu C. Hou Y. Su R. Luo Y. Dou L. Yang Z.. (2022). Effect of dietary probiotics supplementation on meat quality, volatile flavor compounds, muscle fiber characteristics, and antioxidant capacity in lambs. Food Sci. Nutr. 10, 2646–2658. doi: 10.1002/fsn3.2869, PMID: 35959277 PMC9361438

[ref25] Ma Q. L. Hamid N. Bekhit A. E. D. Robertson J. Law T. F. (2012). Evaluation of pre-rigor injection of beef with proteases on cooked meat volatile profile after 1day and 21days post-mortem storage. Meat Sci. 92, 430–439. doi: 10.1016/j.meatsci.2012.05.006, PMID: 22682685

[ref26] Mansur A. R. Seo D. Song E. Song N. Hwang S. H. Yoo M.. (2019). Identifying potential spoilage markers in beef stored in chilled air or vacuum packaging by HS-SPME-GC-TOF/MS coupled with multivariate analysis. LWT 112:108256. doi: 10.1016/j.lwt.2019.108256

[ref27] Mansur A. R. Song E. Cho Y. Nam Y. Choi Y. Kim D.. (2019). Comparative evaluation of spoilage-related bacterial diversity and metabolite profiles in chilled beef stored under air and vacuum packaging. Food Microbiol. 77, 166–172. doi: 10.1016/j.fm.2018.09.006, PMID: 30297047

[ref28] Marino R. Albenzio M. Della Malva A. Santillo A. Loizzo P. Sevi A. (2013). Proteolytic pattern of myofibrillar protein and meat tenderness as affected by breed and aging time. Meat Sci. 95, 281–287. doi: 10.1016/j.meatsci.2013.04.009, PMID: 23743033

[ref29] Nychas G. E. Skandamis P. N. Tassou C. C. Koutsoumanis K. P. (2008). Meat spoilage during distribution. Meat Sci. 78, 77–89. doi: 10.1016/j.meatsci.2007.06.020 22062098

[ref30] Pavlidis D. E. Mallouchos A. Ercolini D. Panagou E. Z. Nychas G. E. (2019). A volatilomics approach for off-line discrimination of minced beef and pork meat and their admixture using HS-SPME GC/MS in tandem with multivariate data analysis. Meat Sci. 151, 43–53. doi: 10.1016/j.meatsci.2019.01.003, PMID: 30685510

[ref31] Pierson C. J. (1976). Effect of postmortem aging time and temperature on pH, tenderness and soluble collagen fractions in bovine longissimus muscle. J. Anim. Sci. 43, 1206–1210.

[ref32] Resconi V. C. Escudero A. Beltrán J. A. Olleta J. L. Sañudo C. Mar Campo M. D. (2012). Color, lipid oxidation, sensory quality, and aroma compounds of beef steaks displayed under different levels of oxygen in a modified atmosphere package. J. Food Sci. 77, S10–S18. doi: 10.1111/j.1750-3841.2011.02506.x, PMID: 22182210

[ref33] Saraiva C. Oliveira I. Silva J. A. Martins C. Ventanas J. García C. (2014). Implementation of multivariate techniques for the selection of volatile compounds as indicators of sensory quality of raw beef. J. Food Sci. Technol. 52, 3887–3898. doi: 10.1007/s13197-014-1447-y, PMID: 26028774 PMC4444891

[ref34] Song S. Zhang X. Hayat K. Liu P. Jia C. Xia S.. (2011). Formation of the beef flavour precursors and their correlation with chemical parameters during the controlled thermal oxidation of tallow. Food Chem. 124, 203–209. doi: 10.1016/j.foodchem.2010.06.010

[ref35] Tansawat R. Maughan C. A. J. Ward R. E. Martini S. Cornforth D. P. (2013). Chemical characterisation of pasture- and grain-fed beef related to meat quality and flavour attributes. Int. J. Food Sci. Technol. 48, 484–495. doi: 10.1111/j.1365-2621.2012.03209.x

[ref36] Utama D. T. Lee S. G. Baek K. H. Jang A. Pak J. I. Lee S. K. (2018). Effects of high-pressure processing on taste-related ATP breakdown compounds and aroma volatiles in grass-fed beef during vacuum aging. Asian Australas J. Anim. Sci. 31, 1336–1344. doi: 10.5713/ajas.17.0677, PMID: 29531191 PMC6043434

[ref37] Vilar E. G. O'Sullivan M. G. Kerry J. P. Kilcawley K. N. (2022). Volatile organic compounds in beef and pork by gas chromatography-mass spectrometry: a review. Sep. Sci. Plus 5, 482–512. doi: 10.1002/sscp.202200033

[ref38] Wahlgren N. M. (1994). Effect of rigor temperature on muscle shortening and tenderisation of restrained and unrestrained beef m. longissimus thoracicus et lumborum. Meat Sci. 51, 61–72. doi: 10.1016/s0309-1740(98)00098-9 22061537

[ref39] Yang X. Zhang Y. Zhu L. Han M. Gao S. Luo X. (2016). Effect of packaging atmospheres on storage quality characteristics of heavily marbled beef longissimus steaks. Meat Sci. 117, 50–56. doi: 10.1016/j.meatsci.2016.02.030, PMID: 26946476

[ref40] Yang X. Zhu L. Zhang Y. Liang R. Luo X. (2018). Microbial community dynamics analysis by high-throughput sequencing in chilled beef longissimus steaks packaged under modified atmospheres. Meat Sci. 141, 94–102. doi: 10.1016/j.meatsci.2018.03.010, PMID: 29606393

[ref41] Yim D. Jo C. Kim H. C. Seo K. S. Nam K. (2016). Application of electron-beam irradiation combined with aging for improvement of microbiological and physicochemical quality of beef loin. Korean J. Food Sci. Anim. Resour. 36, 215–222. doi: 10.5851/kosfa.2016.36.2.215, PMID: 27194930 PMC4869548

[ref42] Zhu M. J. Mendonca A. Ahn D. U. (2004). Temperature abuse affects the quality of irradiated pork loins. Meat Sci. 67, 643–649. doi: 10.1016/j.meatsci.2004.01.005, PMID: 22061814

[ref43] Zhu Q. Zhang S. Wang M. Chen J. Zheng Z. P. (2016). Inhibitory effects of selected dietary flavonoids on the formation of total heterocyclic amines and 2-amino-1-methyl-6-phenylimidazo[4,5-b]pyridine (PhIP) in roast beef patties and in chemical models. Food Funct. 7, 1057–1066. doi: 10.1039/c5fo01055a, PMID: 26781038

